# Supplementary material for: Femtosecond Laser-Induced Nano-Joining of Volatile Tellurium Nanotube Memristor
Source: Nanomaterials (Basel). 2023 Feb 21;13(5):789. doi: 10.3390/nano13050789 (PMC10005240; doi:10.3390/nano13050789)
Supplement: Supplementary file 1 [file nanomaterials-13-00789-s001.zip › nanomaterials-2195800-supplementary.pdf]

nanomaterials

Supplementary Materials

Femtosecond Laser-Induced Nano-Joining of Volatile Tellurium Nanotube Memristor

Yongchao Yu [1,2], Pooran Joshi [3], Denzel Bridges [1], David Fieser [1] and Anming Hu [1,*]

1 Department of Mechanical, Aerospace and Biomedical Engineering, University of Tennessee Knoxville, 1512 Middle Drive, Knoxville, TN 37996, USA
2 School of Mechanical and Aerospace Engineering, Nanyang Technological University, 50 Nanyang Ave., Singapore 639798, Singapore
3 Oak Ridge National Lab, 1 Bethel Valley Rd., Oak Ridge, TN 37831, USA
* Correspondence: ahu3@utk.edu

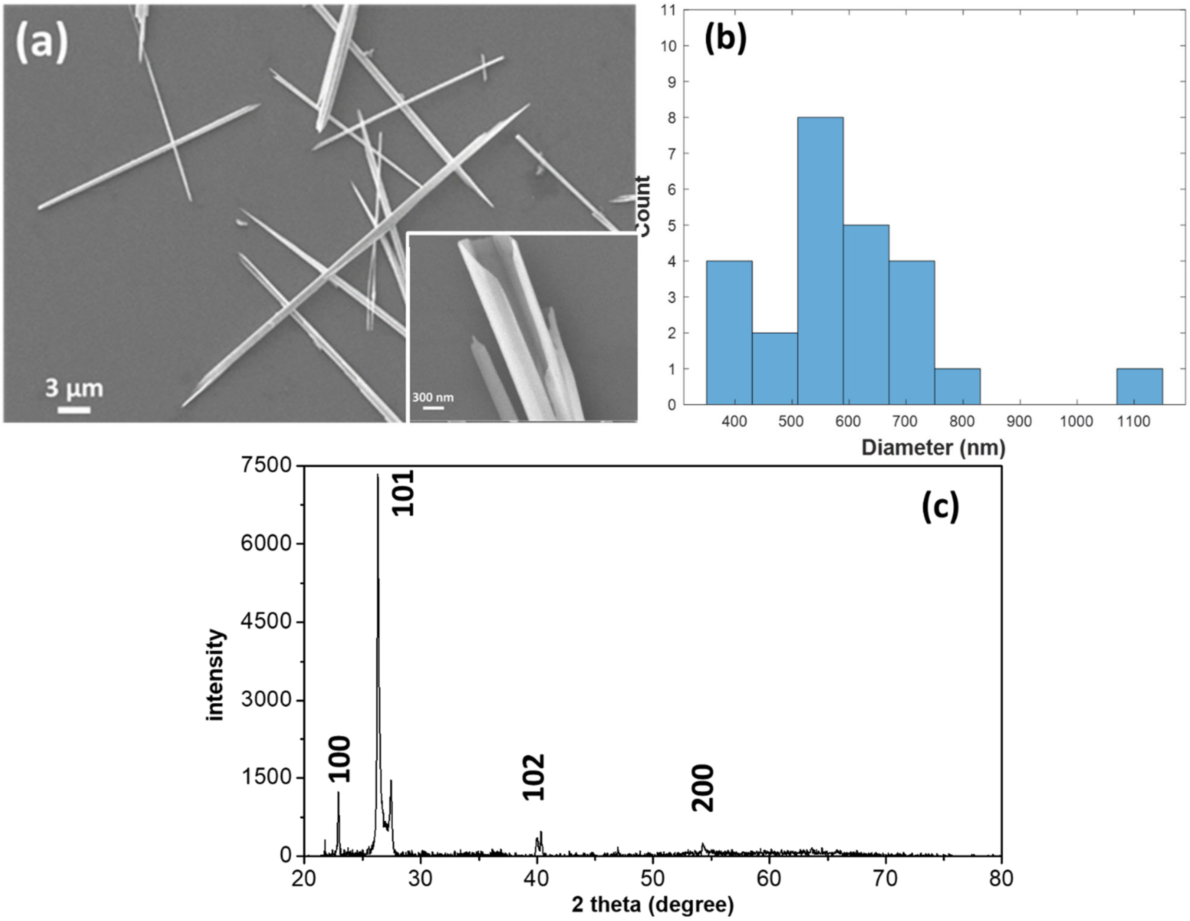

Figure S1. SEM images of (**a**) synthesized Te nanotubes and an enlarged SEM image of Te nanotubes in the inset to Fig. s1(**a**); (**b**) the distribution of nanotube's diameter, and (**c**) XRD analyses for synthesized Te nanotubes.

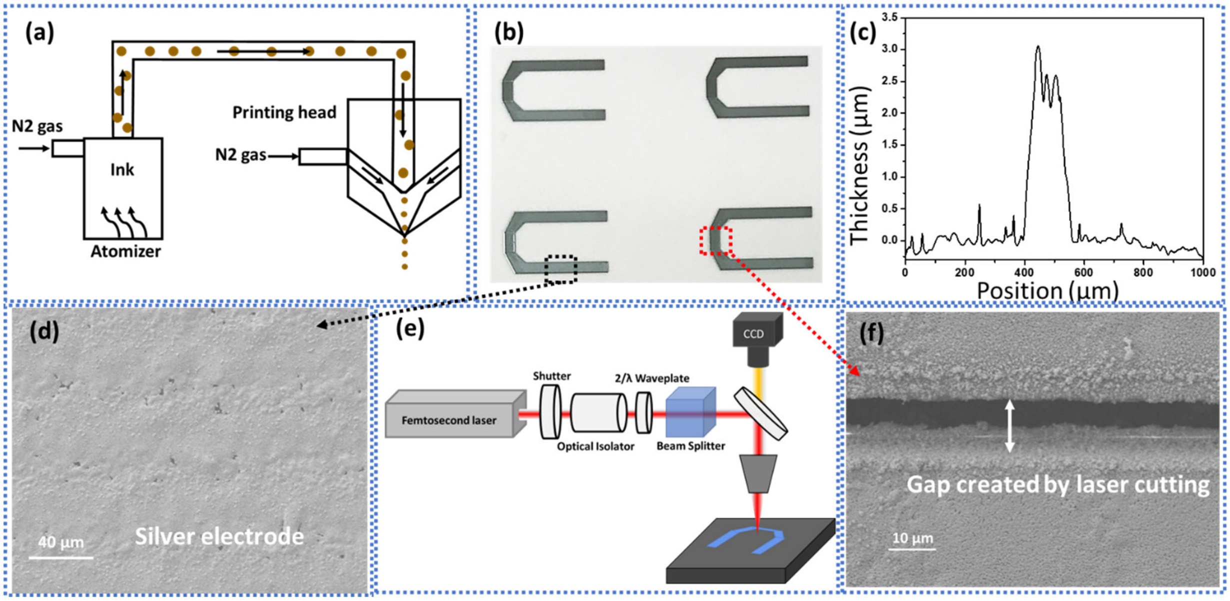

Figure S2. (**a**) Schematic of the aerosol jet printing process, (**b**) an image of silver electrodes printed by aerosol jet printing, (**c**) the thickness measurement result for the silver electrode, (**d**) an SEM image of printed silver electrode's surface after curing(**e**) the schematic of the laser-proccing setup, and (**f**) an SEM image of the gap created by laser cutting process.
